# Inferring nonneutral evolution from contrasting patterns of polymorphisms and divergences in different protein coding regions of enterovirus 71 circulating in Taiwan during 1998-2003

**DOI:** 10.1186/1471-2148-10-294

**Published:** 2010-09-25

**Authors:** Hurng-Yi Wang, Kao-Chien Tsao, Chia-Hung Hsieh, Li-Min Huang, Tzou-Yien Lin, Guang-Wu Chen, Shin-Ru Shih, Luan-Yin Chang

**Affiliations:** 1 Institute of Clinical Medicine, National Taiwan University, Taipei, Taiwan; 2 Department of Medical Biotechnology, Chang Gung University, Taoyuan, Taiwan; 3 Department of Pediatrics, National Taiwan University Hospital, National Taiwan University, 7 Chung-Shan South Road, Taipei 100, Taiwan; 4 Department of Pediatrics, Chang Gung Memorial Hospital, Chang Gung University College of Medicine, Taoyuan, Taiwan; 5 Department of Computer Science and Information Engineering, Chang Gung University, Taoyuan, Taiwan

## Abstract

**Background:**

Enterovirus (EV) 71 is one of the common causative agents for hand, foot, and, mouth disease (HFMD). In recent years, the virus caused several outbreaks with high numbers of deaths and severe neurological complications. Despite the importance of these epidemics, several aspects of the evolutionary and epidemiological dynamics, including viral nucleotide variations within and between different outbreaks, rates of change in immune-related structural regions vs. non-structural regions, and forces driving the evolution of EV71, are still not clear.

**Results:**

We sequenced four genomic segments, i.e., the 5' untranslated region (UTR), VP1, 2A, and 3C, of 395 EV71 viral strains collected from 1998 to 2003 in Taiwan. The phylogenies derived from different genomic segments revealed different relationships, indicating frequent sequence recombinations as previously noted. In addition to simple recombinations, exchanges of the P1 domain between different species/genotypes of human enterovirus species (HEV)-A were repeatedly observed. Contrasting patterns of polymorphisms and divergences were found between structural (VP1) and non-structural segments (2A and 3C), i.e., the former was less polymorphic within an outbreak but more divergent between different HEV-A species than the latter two. Our computer simulation demonstrated a significant excess of amino acid replacements in the VP1 region implying its possible role in adaptive evolution. Between different epidemic seasons, we observed high viral diversity in the epidemic peaks followed by severe reductions in diversity. Viruses sampled in successive epidemic seasons were not sister to each other, indicating that the annual outbreaks of EV71 were due to genetically distinct lineages.

**Conclusions:**

Based on observations of accelerated amino acid changes and frequent exchanges of the P1 domain, we propose that positive selection and subsequent frequent domain shuffling are two important mechanisms for generating new genotypes of HEV-A. Our viral dynamics analysis suggested that the importation of EV71 from surrounding areas likely contributes to local EV71 outbreaks.

## Background

Since its initial isolation in California in 1969 [1,2], enterovirus (EV) 71 was identified to have caused outbreaks of severe neurological disease in Australia, Europe, Asia, and the Americas [3]. Several epidemic outbreaks with high mortality rates have occurred in Bulgaria in 1975 with 44 deaths [4], in Hungary in 1978 with 47 deaths [5], and in Malaysia in 1997 with at least 31 deaths [6]. In a 1998 epidemic, Taiwan had 405 severe EV71 cases with 78 deaths. In 2000 to 2002, there were dozens of fatal EV71 cases each year in Taiwan [7,8]. A stage-based management scheme was developed to reduce the number of case fatalities in Taiwan [9], but most survivors of brainstem encephalitis plus cardiopulmonary failure might have neurologic sequelae and impaired cognition [10]. Clinically, EV71 is capable of causing paralytic disease indistinguishable from poliomyelitis due to the poliovirus. After the poliovirus was nearly eradicated by vaccination, EV71 is now considered one of the most important enteroviruses. Its genome consists of a positive single-stranded RNA molecule of approximately 7400 nucleotides long. The RNA is translated to give a polyprotein which is then proteolytically processed to yield the mature structural P1 domain (encoding the capsid proteins VP4, VP3, VP2, and VP1) and non-structural P2 (non-structural proteins) and P3 domains (non-structural proteins and viral replicase).

There are two major EV71 genotypes (B and C) co-circulating worldwide (the EV71 prototype strain BrCr, isolated in 1969, is the only known example of genotype A). Genotypes B and C are subdivided into sub-genotypes B1~B5 and C1~C5, respectively [11-14]. Despite many studies having focused on the molecular epidemiology of EV71 [11,15-19], several aspects of the evolutionary and epidemiological dynamics are still not clear. In particular, most studies of molecular epidemiology focused only on a single region, particularly VP1, without exploring the dynamics at the genomic scale. There are no rigorous measurements of viral variations within an outbreak and among different epidemic seasons. In addition, although the evolutionary rate of VP1 was estimated [11], little is known as to whether there are discrepancies in immune-related structural vs. non-structural regions and in protein-coding vs. non-coding segments of the EV71 genome. Finally, the processes through which new viral genotypes are generated and evolve are largely unknown.

To help resolve these issues, we examined the evolutionary dynamics of EV71 by analyzing 395 viral isolates sampled in 1998 to 2003 in Taiwan. Four genomic regions, including one capsid protein (VP1), two proteases which cleave the viral polypeptide (2A in the P2 domain and 3C in the P3 domain), and the 5'untranslated region (UTR), were sequenced. Phylogenetic relationships of viruses isolated from different outbreaks were analyzed. We investigated the nucleotide diversity and divergence for different genomic segments, which revealed contrasting patterns for different protein coding regions. Based on the above results, we propose a possible scenario of how new EV71 genotypes emerge and evolve. Finally, we estimated the evolutionary rate and population dynamics of EV71.

## Results

The complete VP1, 2A, and 3C and partial 5'UTR regions of 395 EV71 and 6 coxsackievirus A 16-like (CA16-like) strains isolated in Taiwan in 1998 to 2003 were determined. The aligned lengths of VP1, 2A, and 3C were 891, 450, and 549 nucleotides, respectively. The aligned 5'UTR region including indels was 561 nucleotides long, which spans 151 to 701 nucleotides of EV71-A (BrCr, U22521). Therefore, 2451 nucleotides were sequenced from each of the 401 viral isolates. The strains examined in this study are listed in Additional file 1, Table S1, with the year of isolation and associated clinical symptoms, if known. According to sequence similarities of VP1, genotypes of the recovered viral strains are listed in Table 1. As previously reported [19], EV71-C2 was the major etiologic group in 1998, and EV71-B4 became predominant during the period from 2000 to 2003. Our large-scale survey also revealed that in addition to the major genotype, there were genotypes with very low frequencies co-circulating each year. For example, in addition to the previously reported EV71-C2 and -B4, EV71-C1 and CA16-like were also recovered in 1998. In addition, genotype C2 was found in 1999, and genotypes C4 and CA16-like were discovered in 2001.

**Table 1:** List of viral strains based on the VP1 region collected from 1998 to 2003

	EV71-C1	EV71-C2	EV71-C4	EV71-B4	CA16-like
1998	1	104		7	3
1999		1		4	
2000				61	
2001			1	144	3
2002				65	
2003				7	

### Phylogeny and recombination of EV71

**Figure 1:**
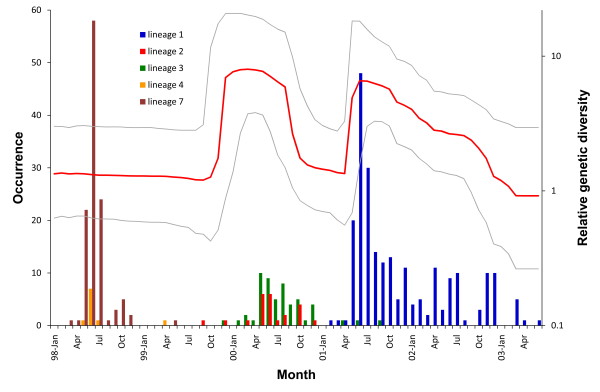
**Occurrences (left y-axis) and temporal changes in the relative genetic diversity (right y-axis) of major enterovirus (EV) 71 lineages each month**. Occurrences of major lineages, lineages 1~4 (EV71-B4) and lineage 7 (EV71-C2), are shown as a bar plot. The lines represent median (red line) and 95% highest posterior densities (HPDs; gray line) of the Bayesian skyline plot derived from VP1 of lineages 1~3.

Within each segment, the homoplasy test [20], GENECONV [21], and GARD [22] failed to identify any recombination event for either within- or between-genotype comparisons. In order to test for possible recombinations between sequenced regions, we combined the four segments for further analysis. The concatenated dataset was divided into 10 lineages at the 1% level of divergence. According to sequence similarities of VP1, genotype B4 included four lineages (1 to 4); genotype C contained three lineages, i.e., lineages 5 (genotype C4), 6 (C1), and 7 (C2); and CA16-like had two lineages, 8 and 9. The last lineage which contained only one viral isolate, TW-2051-98, was identified as a recombinant of lineages 4 and 7 (*p *< 0.01; KH test after GARD and homoplasy test) with the breaking point located between 2A and 3C, and thus it was excluded from further analysis (Additional file 2, Figure S1). No other recombination was detected within any lineage. The occurrences of major lineages from 1998 to 2003 are given in Figure 1. With the exception of 2000, EV71 outbreaks in each season contained only one major lineage. In addition, each lineage was unique to that sampled season with no recurrence in later outbreaks. For example, lineage 4 was from 1998 and 1999, lineages 2 and 3 contained viral strains recovered mainly from 2000, and lineage 1 included viruses from 2001 to 2003.

**Figure 2:**
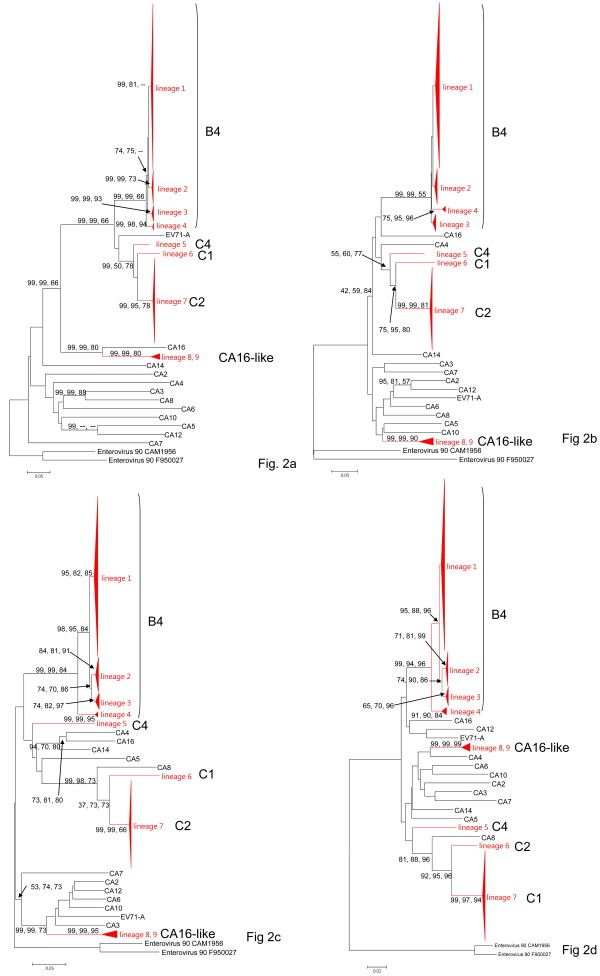
**Neighbor-joining (NJ) trees showing the phylogenetic relationships among human enterovirus (HEV)-A and enterovirus (EV) 71 isolates**. The trees were constructed based on the nucleotide substitution model determined by the Akaike information criterion (AIC) using Modeltest 3.06 from the alignment of VP1 (A), 2A (B), 3C (C), and the 5' untranslated region (UTR) (D), respectively. Numbers, from left to right, close to the nodes are percentages of bootstrap support values of > 70% by the NJ and maximum-likelihood (ML) methods and percentages of support by Bayesian Inference (BI). The height and base length of the red triangles represent the sequence diversity and number of sequences, respectively, within each lineage. Dashes (--) indicate that the nodes were not supported by the method. Numbers of sequences in lineages 1, 2, 3, 4, 7, 8, and 9 are 216, 20, 44, 8, 105, 3, and 3, respectively.

Phylogenetic relationships of viral strains derived from different genomic regions are given in Figure 2 which only shows abridged phylogenies. The complete phylogenetic trees are available in Additional file 3, Figure S2. With the exception of the 2A region, phylogenies derived from different regions, including the 5'UTR, VP1, and 3C, support monophyletic origins of the different lineages (> 70% bootstrap replicates) in most cases, further supporting no recombination existing between them. Since essentially identical relationships of different EV71 lineages were yielded by the maximum-likelihood (ML), neighbor-joining (NJ), and Bayesian inference (BI) methods, we only present the phylogenetic trees constructed by NJ method with nodal supports derived from NJ, ML, and BI analyses given at the nodes.

In the VP1 region, EV71-B4 recovered from different years closely agreed with the phylogenetic positions. Lineage 4 was at the base of the B4 clade followed by lineages 3, 2, and 1. A monophyletic origin of genotype C, including lineages 5, 6, and 7 was strongly supported by the NJ method but with only a 50% bootstrap value in the ML method (Figure 2a). These three lineages together with EV71-A and -B4 formed a monophyletic group with a high bootstrap value (99%). CA16 and its related strain, CA16-like, formed a sister group to EV71. In the 2A region (Figure 2b), all above mentioned lineages are still formed but with low bootstrap support. CA16 did not form a sister group to lineages 8 and 9 as shown in Figure 2a. In the 3C region, lineages 5, 6, and 7 were not clustered together as in Figure 2a and 2b. Instead, lineages 6 and 7 were a sister group to CA8 with 99% bootstrap support in both the ML and NJ methods and 73% probability for BI analysis (Figure 2c). Furthermore, CA16 was not a sister group to lineages 8 and 9. The former was clustered with CA4, and the latter pair was clustered at the bottom of the tree with CA2, CA3, CA6, CA10, CA12, and EV71-A (with 99% bootstrap support in both the ML and NJ trees and 73% probability for BI analysis). In the NJ tree constructed using the 5'UTR, lineages 6 and 7 together were a sister group to CA8 (Figure 2d).

**Figure 3:**
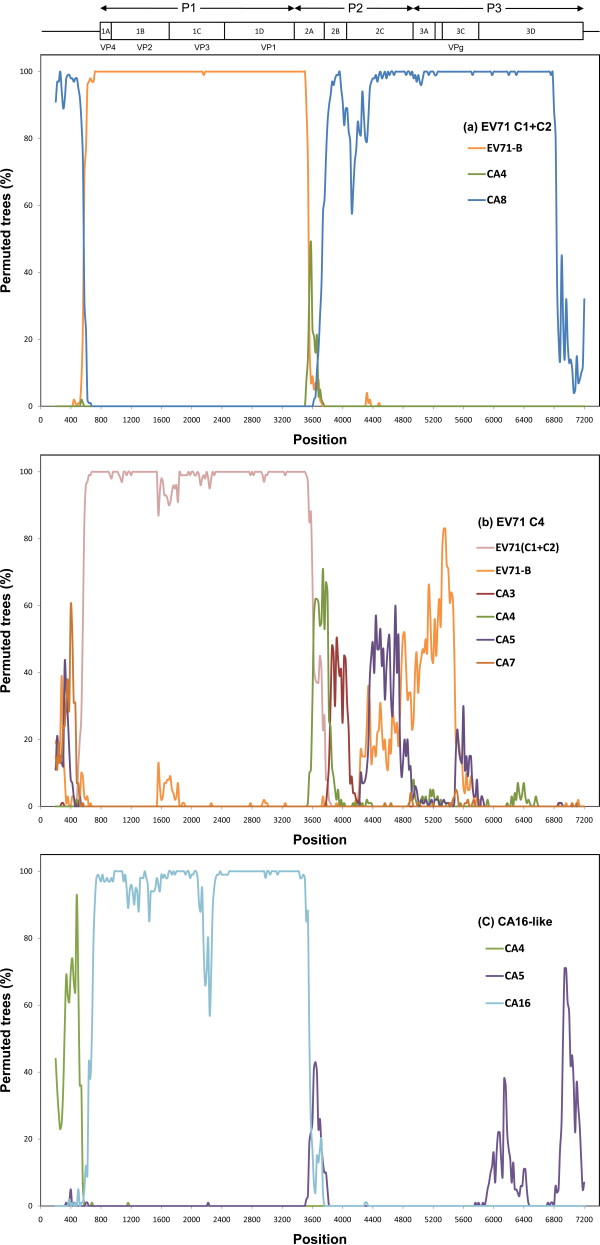
**Identification of recombined regions between different species of human enterovirus (HEV)-A**. The upper panel shows the genomic structure of enterovirus (EV) 71. The results from a bootscan analysis indicated the likelihood of recombination of (a) EV71-C1+C2, (b) EV71-C4, and (c) CA16-like with their potential parental species/genotype. All species listed in "Methods" were used, but only species or groups with > 50% phylogenetic relatedness are shown. EV71-C1+C2 (DQ452074, AF119795, and AF176044); EV71-C4 (AF302996 and AY465356); CA16-like (AF177911 and AY790926); and EV71-B (U22522, AF352027, and AF316321).

Inconsistent phylogenies derived from different genomic regions imply frequent recombinations between different species of human enterovirus species (HEV)-A as previously noted [23]. In Figure 2, lineages 5 and 6 clustered tightly with CA8 in the 3C and 5'UTR regions, but they were recognized as EV71 genotype C (C1 and C2, respectively) based on the VP1 sequences. It is likely that genotypes C1 and C2 were generated by acquiring the structural domain of ancestral EV71. We further used the bootscan program to detect the region of possible recombination. When EV71-C1 and -C2 were compared against different viruses of HEV-A, two recombination events between CA8 and EV71 (either genotype A or B or both combined) in the 5'UTR (within nucleotide 400~600) and 2A regions (within nucleotide 3400~3600) were clearly demonstrated (Figure 3a). Other examples of sequence acquisition were provided by the analyses of the EV71-C4 (Figure 3b) and CA16-like (Figure 3c) viral strains, the structural domains of which only resembled those of EV71-C1+C2 and CA16, respectively. Interestingly, in the above three cases, the break points of recombination were all located at the 3' end of the 5'UTR and 2A regions which neatly covers the entire structural (= P1) domain of the enterovirus.

### Nucleotide diversity and divergence

Average numbers of mutation per site for each year are given in Table 2. We focused only on the major genotypes of each year, including EV71-C2 from 1998 and EV71-B4 from 2000 to 2002. The nucleotide polymorphisms (θ) of the 5'UTR in different years were 1.52%~2.46% which were significantly lower than those of synonymous sites (θ_s_) of the three protein coding regions (*p *= 0.014; Mann-Whitney test). In addition to low polymorphism, the 5'UTR had higher proportions of singletons than did synonymous sites of protein coding regions. Proportions of non-singletons polymorphisms, i.e., the number of mutations which occurred more than once divided by the number of total mutations, in the 5'UTR was significant lower than those of synonymous sites of VP1 (*p *= 0.021), 2A (*p *= 0.041), and 3C (*p *= 0.041). θ_s _values derived from different coding regions ranged 8.11%~10.03% for VP1, 8.37%~12.33% for 2A, and 9.34%~11.38% for 3C, and the differences among them were not significant (*p *> 0.05). Nevertheless, nucleotide polymorphisms at nonsynonymous sites (θ_a_) of VP1, ranging 0.19%~0.40%, were significantly lower than those of 2A and 3C which were 0.46%~0.97% (*p *= 0.014). Consequently, the ratios of nonsynonymous to synonymous nucleotide polymorphisms (θ_a_/θ_s_) of VP1, ranging 0.024~0.04, were 2~3-times smaller than those of 2A and 3C (*p *= 0.014). We also analyzed differences in ratios of nonsynonymous (N) to synonymous (S) mutations among the three coding regions. The N/S ratios for VP1, at 0.08~0.14, were smaller than those of 2A (*p *= 0.001) and 3C (*p *= 0.014). The difference between VP1 and 2A became insignificant after the singletons were removed.

**Table 2:** Polymorphisms in coding and non-coding regions of enterovirus (EV) 71 derived from viruses collected in different years.

		VP1	2A	3C	5'UTR
**Genotype**	**Year**	**θ**_**s**_			**θ**
EV71-C2	1998	8.11% (86, 35)	9.74% (50, 19)	9.70% (61, 24)	1.52% (41, 7)
EV71-B4	2000	10.03% (94, 49)	10.69% (49, 28)	9.55% (53, 18)	1.54% (39, 12)
EV71-B4	2001	9.25% (101, 54)	12.33% (67, 28)	11.38% (76, 46)	2.46% (66, 11)
EV71-B4	2002	8.74% (83, 47)	8.37% (39, 28)	9.34% (53, 35)	1.84% (49, 23)
Average		8.87%	10.28%	9.99%	1.84%
*p *value (vs. VP1)			NS	NS	
*p *value (vs. UTR)		0.014	0.014	0.014	
*p *value (vs. UTR)^A^		0.021	0.041	0.041	
		θ_a_			
EV71-C2	1998	0.19% (7, 2)	0.65% (12, 2)	0.57% (13, 6)	
EV71-B4	2000	0.40% (13, 7)	0.66% (11, 4)	0.80% (16, 6)	
EV71-B4	2001	0.29% (11, 5)	0.97% (19, 5)	0.46% (11, 8)	
EV71-B4	2002	0.21% (7, 3)	0.54% (9, 1)	0.49% (10, 4)	
Average		0.25%	0.70%	0.58%	
*p *value (vs. VP1)			0.014	0.014	
		θ_a_/θ_s _(N/S)^B^			
EV71-C2	1998	2.35% (0.08, 0.06)	6.69% (0.24, 0.11)	5.87% (0.21, 0.25)	
EV71-B4	2000	3.99% (0.14, 0.14)	6.19% (0.22, 0.14)	8.35% (0.30, 0.33)	
EV71-B4	2001	3.11% (0.11, 0.09)	7.87% (0.28, 0.18)	4.06% (0.14, 0.17)	
EV71-B4	2002	2.43% (0.08, 0.06)	6.41% (0.23, 0.04)	5.25% (0.19, 0.11)	
Average		2.82% (0.10, 0.09)	6.79% (0.24, 0.12)	5.81% (0.21, 0.22)	
*P*-value(versus VP1)			0.014 (0.001, NS)	0.014 (0.014, 0.021)	

Numbers in parentheses are total number of mutations and number of mutations without singletons, respectively.

*p *values are from the Mann-Whitney one-tailed test.

NS, not significant (*p *> 0.05).

A, Comparison of proportions of non-singleton mutations between different genomic regions.

B, Ratios of nonsynonymous (N) to synonymous (S) mutations.

Ka and Ks values between EV71 and HEV-A are shown in Table 3. Since the outgroup of EV71 was difficult to identify, other than for the VP1 region (Figure 2), results are expressed as the average divergence between EV71 and different species of HEV-A. Divergences of the 5'UTR (K), ranging 0.116~0.139, were much lower than Ks values of the protein coding regions, which ranged 1.1~1.8 (Table 3). The Ka of VP1 between EV71 and other HEV-As, being 0.384 for EV71-B4 and 0.383 for EV71-C2, were higher than those of 2A and 3C which were 0.038~0.057. As a result, rates of protein evolution after calibration by the neutral mutation rate (as presented by Ka/Ks) for VP1, at 0.211 and 0.221, were much higher than those of 2A and 3C, at 0.023~0.034. Small Ka and Ka/Ks values between EV71-B4 and -C2, at 0.016 and 0.015, respectively, indicated that among different genotypes of EV71, VP1 was extremely conserved.

**Table 3:** Divergences in coding and non-coding regions between different genotypes and species of human enterovirus (HEV)-A

	Ks			K
	VP1	2A	3C	UTR
EV71-B4 vs. EV71-C2	1.102	1.320	1.701	0.137
EV71-C2 vs. HEV-A	1.813	1.618	1.546	0.139
EV71-B4 vs. HEV-A	1.734	1.654	1.690	0.116
				
	**Ka**			
EV71-B4 vs. EV71-C2	0.016	0.027	0.055	
EV71-C2 vs. HEV-A	0.383	0.043	0.039	
EV71-B4 vs. HEV-A	0.384	0.038	0.057	
				
	**Ka/Ks**			
EV71-B4 vs. EV71-C2	0.015	0.020	0.032	
EV71-C2 vs. HEV-A	0.211	0.027	0.025	
EV71-B4 vs. HEV-A	0.221	0.023	0.034	

### Tests for positive selection

Since Ks was saturated, the Ka/Ks ratio should be interpreted with care. We tested whether different protein coding regions had different selection regimes by HyPhy (see "Methods"). The results of likelihood ratio test (LRT) revealed no sign for codon under positive selection. In addition, compared to VP1, 2A and 3C were under stronger purifying selection (p < 10^-3 ^for VP1 vs. 2A, p < 0.05 for VP1 vs. 3C, and p = 0.91 for 2A vs. 3C). Therefore, higher Ka/Ks ratio in VP1 cannot be solely imputed to the saturation on synonymous sites, which suggests that different evolutionary forces may have dominated different regions of the genome.

Different approaches were applied to test the hypothesis of positive selection in the VP1 region. We first performed site model tests implemented in PAML to search for evidence of positive selection. The results of LRTs for M1a vs. M2a and M7 vs. M8 were both insignificant, suggesting that the evidence of positive selection working on a particular codon was limited. Using model M2a, we estimated that 98% of the codons were subject to negative selection with an average Ka/Ks ratio of 0.01, 2% had evolved under near neutrality with Ka/Ks ≈ 1, and there was no site with Ka/Ks > 1. We also used branch-site model A to test for positive selection on individual sites along specific lineages (foreground branches). Different lineages were sequentially assigned as foreground branches to test against the null model with Ka/Ks = 1. This test also failed to detect a signature of positive selection on specific branches. PAML also implemented a Suzuki and Gojobori-style [24] method to test the effects of selection at individual sites with changes according to ancestral states reconstructed by the likelihood method. The results suggested that 98% of the sites were under negative selection, and that only 2% had Ka/Ks values of > 1, but they were all insignificant. Similar results were noted in the analysis of the poliovirus surface protein [25]. Therefore, within enterovirus, evidence of positive selection at individual sites was limited.

Because synonymous substitutions were saturated (Ks > 1; Table 3), the standard Ka/Ks analysis might not be a suitable statistical test for positive selection. We, therefore, performed computer simulations. In each of 10,000 simulations, the number of nucleotide changes was drawn from a Poisson distribution until Ks was equal to the average Ks between EV71 and HEV-A (see "Methods"). Ratios of nonsynonymous (N) to synonymous (S) mutations were chosen according to the observed N/S ratios in the polymorphisms, which were 0.10, 0.24, and 0.21 for VP1, 2A, and 3C, respectively (Table 2). The objective of this test is to detect natural selection in the sequences. If the selection intensity is constant over the evolutionary time scale, the ratio of N to S fixations should be equal to the ratio of N to S polymorphisms. The pattern will differ, however, when other forms of selection act on nonsynonymous mutations. Positive selection will increase the divergence relative to polymorphisms at selected sites, whereas negative selection is expected to result in the opposite pattern.

**Figure 4:**
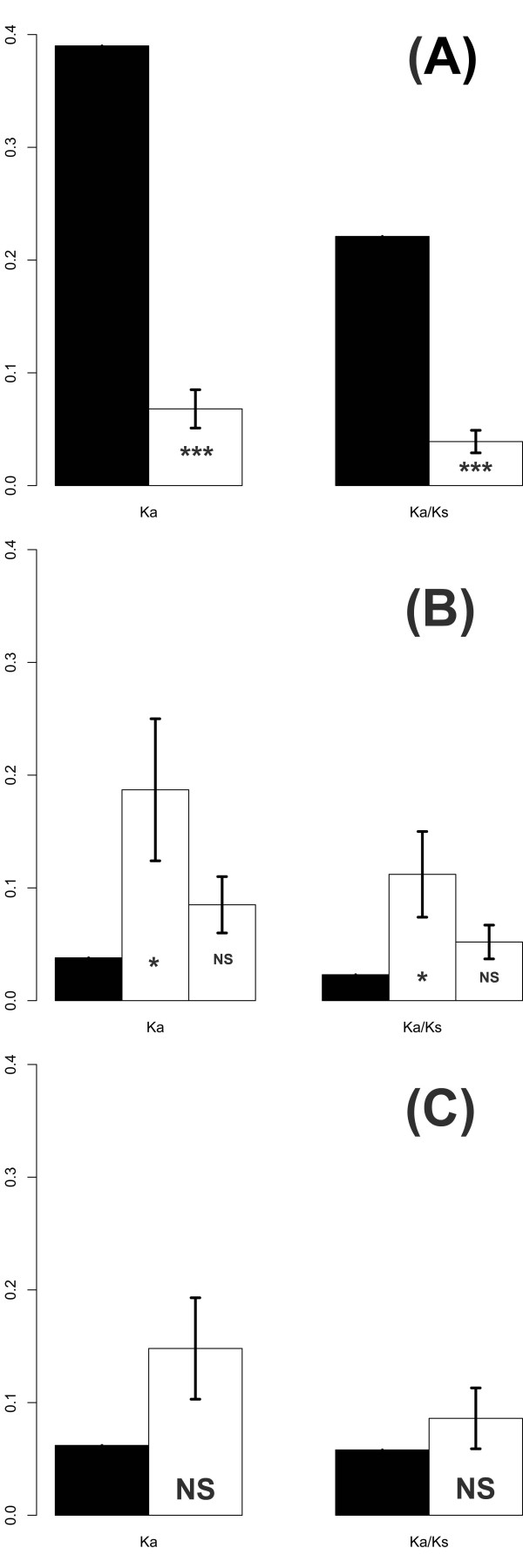
**Observed (filled box) and simulated (open box) Ka and Ka/Ks values for VP1 (A), 2A (B), and 3C (C), respectively, between enterovirus (EV) 71-B4 and human enterovirus(HEV)-A**. Simulated Ks values were set equal to the observations. The proportions of nonsynonymous (N) and synonymous (S) mutations were determined according to the observed N/S ratios in polymorphisms which were 0.10 for VP1, 0.24 and 0.12 for 2A, and 0.21 for 3C (see Table 2 and text for details). The error bar represents 1 standard deviation. *p *values were determined by 10,000 simulations. * *p *< 0.05; *** *p *< 0.001; NS, not significant (*p *> 0.05).

Simulations were done for both EV71-C2 and B4, and the results were essentially the same. For illustrative purposes, we only present the results of genotype B4 (Figure 4). For VP1, the observed Ka and Ka/Ks values were significantly higher than the simulated results (*p *< 10^-3^; Figure 4a), indicating accelerated fixation of amino acid replacement substitutions. Such a pattern was reversed for 2A and 3C (Figure 4b, c). In the 3C region, the observed Ka and Ka/Ks values were lower than the predictions, but the difference was insignificant. Significantly lower Ka and Ka/Ks values in the simulations than in observations for 2A suggest the effect of negative selection preventing deleterious mutations from becoming fixed. Deleterious mutations are usually at a low frequency and inflate the N/S ratio of polymorphisms. This problem can be partially overcome by removing rare mutations from the sample [26]. The N/S ratio of 2A was largely reduced, from 0.24 to 0.12 (Table 2), after singletons were removed. We then reset the N/S ratio from 0.24 to 0.12 for 2A in the simulation, and differences in Ka and Ka/Ks between observations and predictions became non-significant (Figure 4b). In short, when singletons were not counted, 2A and 3C showed good agreement with the neutral mutation hypothesis.

Reduced levels of polymorphism and divergence in the 5'UTR resemble general patterns of protein evolution and indicate that the 5'UTR was either under selection or was subjected to a lower mutation rate than were synonymous sites. A framework, proposed by McDonald and Kreitman (MK), of comparing levels of polymorphism within and divergence between different viruses can distinguish neutrality from selection in the sequences [27]. If reduced levels of polymorphism and divergence in the 5'UTR can be explained by a lower mutation rate, the ratio of polymorphisms to divergences should be similar to that for synonymous sites. Although, the MK test was originally developed to detect selection at classes of different sites that have the same evolutionary history, it can also be generalized to consider other classes of selected sites from different regions, including non-coding regions [26], even when they are completely unlinked [28]. We then applied the MK test to the 5'UTR of EV71-C2 vs. CA8 and EV71-C2 vs. -C1 using polymorphisms and divergence in the 3C region as a reference, because CA8 was strongly supported as the outgroup of EV71-C1 and -C2 in these two regions (Figure 2c, d) and because Ks values between CA8 and EV71-C2 at 0.73 and between EV71-C1 and -C2 at 0.42 were not saturated.

We first applied the MK test using all polymorphisms, and the non-significant results suggested a neutral pattern of molecular evolution which seemed to support a low mutation rate in the 5'UTR of HEV-A. Nevertheless, since the 5'UTR exhibited many rare variants (Table 2) which may be deleterious and are likely to be a factor limiting the power to detect selection in the sequences [29], we next removed all singletons from the sequences. Applying this approach revealed a significant excess of divergence in the 5'UTR of EV71-C2 compared to CA8 (*p *= 0.045; Chi-square test) and EV71-C1 (*p *= 0.026; Table 4). This result suggested that a significant fraction of divergence in the 5'UTR was probably driven to fixation by positive selection.

**Table 4:** Polymorphism and divergence in the 5' untranslated region (UTR) and 3C regions of enterovirus (EV) 71-C

	Div-S	Div-UTR	Poly-S	Poly-UTR	**P**^**A**^	**Poly-S**^**B**^	**Poly-UTR**^**B**^	**P**^**C**^
CA8 vs. EV71-C2	63.4	48.7	64	49	0.95	29	10	0.045*
EV71-C1 vs. -C2	31.6	29	64	49	0.60	29	10	0.026*

Div-S, the number of divergent silent changes in 3C after correction by the modified Nei and Gojobori method.

Div-UTR, the number of divergent changes in the 5'UTR after correction by the Juke and Canter method.

Poly-S, the number of polymorphic silent changes in 3C.

Poly-UTR, the number of polymorphic changes in the 5'UTR.

A, Chi-square probability using all polymorphisms.

B, the number of changes excluding singletons.

C, Chi-square probability excluding singleton polymorphisms.

### Population demographics and the rate of change

Evolutionary dynamics, represented by the VP1 region, were estimated using an established Bayesian Markov chain Monte Carlo (MCMC) approach that incorporates the month of viral sampling. In the absence of natural selection, the genetic diversity obtained reflects the change in the effective number of infections over time [30]. Nevertheless, a possible role of selection was demonstrated for VP1 of EV71 in a previous section; the plot was interpreted as a measure of the relative genetic diversity. Changing patterns of genetic diversity clearly showed temporal dynamics of EV71 isolated from 1998 to 2003 (Figure 1). Two peaks, from left to right, respectively represent the outbreak from late 1999 to 2000 (lineages 2 and 3) and the epidemic seasons of 2001 to 2003 (lineage 1). Genetic diversities were largely reduced at the end of each epidemic season demonstrating genetic bottlenecks. Because EV71-C2 (lineage 7) was mainly recovered in 1998 which yielded a large uncertainty in the diversity estimation, we did not include lineage 7 in Figure 1.

For each region, the time to the most recent common ancestor (TMRCA) recovered from different outbreaks was separately estimated (Table 5). For lineage 1, and lineages 2 and 3, the TMRCAs derived from different segments were very close, indicating that within these epidemic seasons, different genomic regions may have shared similar evolutionary histories. For lineage 7, the TMRCAs of four different segments exhibited greater variation than the above three lineages. All the TMRCAs predated the beginning of the outbreaks. For example, the TMRCA of VP1 of lineages 2 and 3, both of which caused the epidemic from late 1999 to 2000, was 1998.6 (i.e., June 1998) with the 95% highest posterior density (HPD) ranging 1997.9~1999.2. The TMRCA of VP1 for viral strains recovered from 2001 to 2003 (lineage 1) was 2000.8 (95% HPD, 2000.5~2001.1). Since our sequences were collected within a relatively short period of time which may have constrained the posterior distribution of that TMRCA, we next included VP1 sequences collected between the 1990 s and 2003 from surrounding areas for TMRCA estimation (see "Methods"). The results shown in Table 5 are close to our original estimations with narrower 95% HPDs. We also performed a Bayesian MCMC analysis using randomly associated sequences and dates. For the VP1 region, the TMRCAs of the generated dataset were 1852 (95% HPD, 1480~1991) for lineage 1 and 1725 (95% HPD, 1063~1985) for lineages 2 and 3. Therefore, our estimation of TMRCAs is sound.

**Table 5:** The time to the most recent ancestor (TMRCA) of different segments of enterovirus 71 derived from this study.

Lineage	Beginning of the outbreak	Genomic region	TMRCA	95% HPD
Lineage 1	2001.2	VP1	2000.8 (2000.8)	2000.5~2001.1 (2000.3~2001.1)
		2A	2000.8	2000.3~2001.1
		3C	2000.8	2000.4~2001.0
		5'UTR	2000.4	1999.9~2000.9

Lineage 2 and 3	1999.8	VP1	1998.6 (1997.8)	1997.9~1999.2 (1997.1~1998.4)
		2A	1998.9	1998.3~1999.4
		3C	1998.8	1998.2~1999.3
		5'UTR	1998.8	1998.1~1999.4

Lineage 7	1998.3	VP1	1997.2 (1997.3)	1995.6~1998.1 (1996.7~1997.8)
		2A	1998.1	1997.4~1998.2
		3C	1997.3	1984.4~1998.4
		5'UTR	1998.0	1997.7~1998.2

Numbers in parentheses are TMRCA estimated with seqeunces from surrounding areas (see text for details)

HPD, highest posterior density.

The evolutionary rates of different genomic segments of EV71-B4 recovered in this study between 1998 and 2003 were estimated. The overall rates of change estimated by the linear regression method for the VP1, 2A, 3C, and 5'UTR regions were 4.6 × 10^-3^, 6.8 × 10^-3^, 7.1 × 10^-3^, and 5.3 × 10^-3 ^per site per year (/site/year), respectively. These values are generally in good agreement with those derived from the Bayesian MCMC method which were 5.7 × 10^-3^, 6.7 × 10^-3^, 6.3 × 10^-3^, and 5.5 × 10^-3^/site/year for VP1, 2A, 3C, and 5'UTR, respectively (Table 6). In addition, evolutionary rates estimated by the linear regression fell within the 95% HPDs derived from the Bayesian MCMC method. Similar values derived from different approaches indicated that our estimates were authentic. For the protein coding regions, approximately 70%~90% of all changes were synonymous changes (Table 2). We then estimated the rate of change for the third codon position by Bayesian MCMC and on synonymous sites by the linear regression method (see "Methods"). The evolutionary rate for third codon position for VP1, 2A, and 3C were 1.48 × 10^-2^, 1.66 × 10^-2^, and 1.51 × 10^-2 ^(/site/year), respectively. Because not all changes on the third codon position are synonymous, these values are smaller than synonymous mutation rates which were 2.2 × 10^-2^, 3.5 × 10^-2^, and 3.2 × 10^-2^/site/year for VP1, 2A, and 3C, respectively. For the 2A and 3C regions, synonymous mutation rates are close to that of the poliovirus (3.96 × 10^-2^/site/year) [31], but higher than estimates by Hanada et al. (1.00 × 10^-2^) [32] and Brown et al. (1.35 × 10^-2^) [11] of enterovirus.

**Table 6:** Rates of change in different genomic regions of enterovirus (EV) 71-B4 derived from this study

	Evolutionary rate (changes/site/year)					
**Region**	**By linear regression**				**By Bayesian Markov chain Monte Carlo**	
	**Synonymous sites**	***R***^**2**^	**All sites**	***R***^**2**^	**Third codon position** **(95% HPD)**	**All sites** **(95% HPD)**

VP1	2.2 × 10^-2^	0.36	4.6 × 10^-3^	0.36	1.48 × 10^-2^ (1.43 × 10^-2^~1.54 × 10^-2^)	5.7 × 10^-3^ (4.6 × 10^-3^~6.7 × 10^-3^)
2A	3.5 × 10^-2^	0.70	6.8 × 10^-3^	0.58	1.66 × 10^-2^ (1.54 × 10^-2^~1.74 × 10^-2^)	6.7 × 10^-3^ 5.2 × 10^-3^~8.5 × 10^-3^
3C	3.2 × 10^-2^	0.59	7.1 × 10^-3^	0.54	1.51 × 10^-2^ (1.41 × 10^-2^~1.61 × 10^-2^)	6.3 × 10^-3^ 4.9 × 10^-3^~7.7 × 10^-3^
5'UTR	-	-	5.3 × 10^-3^	0.60	-	5.5 × 10^-3^ 4.1 × 10^-3^~7.0 × 10^-3^

Bayesian Markov chain Monte Carlo was conducted by BEAST v1.4.8 using the GTR+Γ+SRD06 [75] model of nucleotide substitution and strict molecular clock (see Methods for details).

*R*^2^, regression coefficient.

HPD, highest posterior density; UTR, untranslated region.

## Discussion

### Signature of the positive selection of EV71

VP1 was previously demonstrated to have rapidly evolved [1]. The question is whether positive selection needs to be invoked to account for the high rate of amino acid substitutions or whether alternative explanations of past population demographics, relaxation of selective constraints, and elevation of the mutation rate are sufficient. It was argued that accelerated amino acid substitutions can occur if some nonsynonymous mutations are slightly deleterious, and the current effective population size is larger than the long-term effective population size [27]; this is because slightly deleterious mutations that are currently not segregated in the population may have been fixed in the past. A historical reduction in population size will also have generated an excess of amino acid substitutions in divergence [33]. For viruses producing only acute infections, such as EV71 and influenza, the population size (judged by the number of transmissions) exponentially increases during an outbreak and then experiences a dramatic reduction after the epidemic. The population dynamics shown in Figure 1 clearly demonstrate that EV71 experienced dramatic population size changes between the different epidemic seasons. Nevertheless, while positive selection acts on specific target regions, the influence of population demographics affects all regions of the genome. Smaller Ka and Ka/Ks values found in non-structural regions (2A and 3C) than in the structural region (VP1) (Table 3) demonstrated that accelerated amino acid substitutions in the latter are not a genome-wide phenomenon and, thus, are unlikely to solely be the consequence of population size changes. If relaxation of selective constraints is responsible for the rapid amino acid replacement between different viruses, levels of θ_a _and θ_a_/θ_s _in VP1 should have increased accordingly. This is not expected under positive selection because mutations would have short retention times in the population and hence would contribute little to polymorphisms. Significantly lower θ_a _and θ_a_/θ_s _values in VP1 than in 2A and 3C (Table 2) indicate no sign of relaxation in the former. Finally, comparable levels of θ_s _among the three protein coding regions clearly illustrate no significant differences in their underlying mutation rates. Consequently, the rapid evolution of VP1 cannot be reconciled with past population demographics, relaxation of selection, or differences in mutation rates. The contrasting patterns of polymorphism and divergence in VP1, thus, should best be explained by the hypothesis that positive Darwinian selection is responsible for the high rate of amino acid replacements.

Among the three outer surface proteins, VP1, VP2, and VP3, which comprise the outer surface of the viral capsid, serotype-specific immune epitopes are predominantly located in VP1 [34]. Immunization using a recombinant VP1 protein of EV71 was shown to confer protection against lethal EV71 infection in newborn mice, indicating that VP1 contains important antigenic sites [35]. In addition, VP1 plays an important role in cell attachment [36]. It is, therefore, temping to speculate that VP1 is under strong selection by host immune systems which would result in accelerated amino acid changes between different species of HEV-A.

While our simulation results suggest positive selection in the VP1 region, codon-based LRTs failed to detect such a signal. This inconsistency was probably due to the following reasons. First, simulations demonstrated that LRTs are not robust to frequent recombinations [37] which is very common in enteroviruses (Figure 3). Nevertheless, recombination does not create contrasting patterns of polymorphisms and divergences in different genomic regions. Second, as shown in Tables 2 and 3, nucleotide sequences were highly diverged between different species and genotypes of HEV-A, but were very similar within each genotype. It was proposed that highly biased trees might harbor too many changes along the long branches and too few changes along the short branches, leading to a lack of information and low power of the detection method [38]. Tee et al. (2010) applied globally sampled VP1 sequences and identified three putative sites under positive selection [39], suggesting that a more comprehensive sampling may be necessary to identify site under selection.

Couple lines of evidence also suggest that the 5'UTR may be under positive selection. First, after singletons were removed, the results of the MK test in Table 4 suggested an excess of nucleotide substitutions in divergence, implying the action of selection. Second, a higher proportion of singletons in the 5'UTR than in synonymous sites of protein coding regions (Table 2) implied that the effect of selection has kept deleterious mutations at low frequencies. The 5'UTR of EV71 contains six putative stem-loop structures [40]. Stem-loop I is essential for viral RNA synthesis, while stem-loop II-VI comprise the internal ribosome entry site (IRES) necessary for translation initiation [41,42]. The maintenance of these structure elements is critical for viral viability. Mutations which destroy base pairing within the stem are therefore deleterious [42] and will be eliminated or kept in low frequency. On the other hand, compensatory mutations which restore base pairing might have to be fixed at once in order to maintain proper secondary structure. In short, the 5'UTR plays important roles for viral RNA synthesis and translation initiation, and the reduced levels of polymorphism and divergence may be a result of selection.

### Mechanism generating new genotypes

It was proposed that recombination contributed to the emergence of various species/genotypes of enterovirus [43]. Instead of random recombinations, however, we observed a consistent phenomenon of acquiring the P1 domain from different genotypes or species of HEV-A. It is unreasonable to expect that chance alone would generate such a repeating pattern (Figure 3). Frequent sequence shuffling, i.e., acquiring a piece of a nucleotide sequence from different viruses, may have had important impacts on the evolution of enteroviruses. As shown by the signature of positive selection in the VP1 region, it is possible that acquiring the P1 domain from donors which carry rapid and presumably adaptive changes would cause these recipient viral strains to gain immediate advantage over others. Consequently, based on the current molecular genetic analysis and previous phylogenetic studies [23,43], we hypothesized that positive selection and frequent domain shuffling are two important mechanisms for generating new types of enteroviruses.

It must be pointed out that the results in Figure 3 do not themselves provide evidence that these genotypes were generated by recombination between particular viral strains. For example, in the analysis of EV71-C1+C2, when we used either EV71-B or EV71-A, separately, or combined them as one reference group, the results were identical. But when EV71-A and -B were both presented as different reference groups, neither of them showed high relatedness with EV71-C1+C2 in the P1 domain. That is because both EV71-A and -B are equal distances from EV71-C1+C2 in that region (Figure 2a). Therefore, it is possible that EV71-C1+C2 was generated by ancestral sequences of CA8 and EV71 or viral strains very close to them.

### Origin of local outbreaks

**Figure 5:**
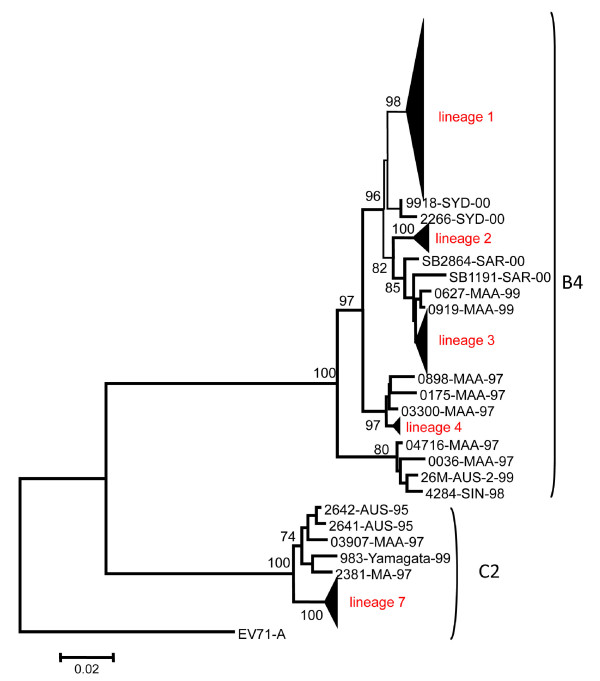
**Phylogenetic tree for the VP1 region of enterovirus (EV) 71 isolates in Taiwan between 1998 and 2003 and from surrounding areas between the 1990 s and 2003. Numbers close to the branches are from 1000 bootstrap replicates (%)**. The two digits at the end of a sequence indicate the year of isolation. Yamagata: Yamagata, Japan; Aus: Australia; SYD: Sydney, Australia; MAA and MA: Malaysia; SAR, Sarawak, Malaysia; Sin: Singapore.

Observations of high viral diversity during epidemic peaks with periodic reductions suggest that either the viruses were maintained in the population and across inter-epidemic intervals or the viruses were imported from other sources as shown in a previous study [13]. Co-circulation of different viral genotypes in extremely low frequencies might be an indication of the former (Table 1). It is possible that these viral strains had been in circulation for some time before the number of cases became large enough to be noted. For example, the viral strains causing the 1999~2000 outbreak were first sampled in September 1999 (1999.75; Figure 1). As the TMRCA estimated for that outbreak ranged 1998.6~1998.9, the above hypothesis suggests that the virus had been circulating for approximately a year before it was sampled. Nevertheless, as EV71 has been rigorously monitored since the 1998 outbreak in Taiwan, it seems unlikely that the virus was able to remain unnoted or did not cause any severe case for almost a year while in circulation. Furthermore, the estimated TMRCAs were all beyond the beginning of the outbreak in which they were sampled (Table 5). To further investigate the phylogenetic origin of different lineages, we acquired EV71 sequences from surrounding areas during the 1990 s to 2003. Phylogenetic relationships of different viral strains is given in Figure 5. Lineage 7 is phylogenetically close to viral strains reported in Malaysia, Australia, and Japan between 1995 and 1999. Lineage 4 from 1998 and 1999 was tightly clustered with the B4 strain from Malaysia in 1997. Lineages 2 and 3 were grouped with viral isolates from Malaysia between 1999 and 2000. Finally, lineage 1 was closest to the B4 strain from Australia in 2000. Figure 5 suggests that EV71 in Taiwan is frequently imported and perhaps moves from and to surrounding areas. Because local viral isolates showed more than 97% identity to their sister group which had been circulating in surrounding areas immediately prior to an outbreak, the importation of viral strains to the local area is the most parsimonious explanation. Both TMRCAs and phylogenetic analyses failed to support the maintenance of viral diversity locally. Alternatively, our results may actually indicate that viral diversities were not maintained in the local area, but were imported from other sources.

Our conclusion of viral importation is not consistent with previous study which suggested that EV71 outbreaks caused by the same subgenotype in different countries may be the result of different lineages and were less likely to have been caused by transmission between countries [39]. This conclusion was drawn from the analysis of VP1 sequences at global scale, nevertheless. It is possible that different lineages are indeed maintained in a large area with occasional movement of viral lineages between continents, while within each area, e.g. east Asia, transmissions between borders may be still common [13].

### Evolutionary rate estimation

Our estimated rates of change for synonymous sites of 2A and 3C were close to that of the poliovirus (3.96 × 10^-2^/site/year) [31], but higher than estimations by Hanada et al. (1.00 × 10^-2^)[32], and Brown et al. (1.35 × 10^-2^)[11] of enterovirus. This discrepancy is reconcilable by distinguishing the mutation rate, which is related to the tempo of change at the population level, from the substitution rate, which reflects long-term evolutionary change. When mutations are selectively neutral, or nearly so, the rate at which they are generated (the mutation rate) should have a simple relation with the rate of fixation (the substitution rate) [44]. Deviation from this neutral expectation can reveal fundamental aspects of evolutionary and biological processes [32,45]. Under negative selection, mildly deleterious mutations can linger in populations, contributing to polymorphisms, but have little chance of fixation [46]. Therefore, the rate of evolutionary change based on population-level estimates would be larger than those based on long-term follow-up studies, as the former includes mutations doomed by elimination. In our simulations, after singletons were removed, the difference in N/S ratios between polymorphisms and divergences became insignificant (region 2A, Figure 4b), indicating the existence of low-frequency deleterious mutations in polymorphisms. The rates of change for the poliovirus [31] and EV71 in the current study were derived in a short period of time and are expected to be close to population-level estimates. It is then not unreasonable that these values are larger than those of previous long-term studies [11,32].

The above scenario can also explain the seemingly counterintuitive observation that the rate of change for the VP1 capsid protein was slower than those of 2A and 3C which have only internal functions (Table 6). It is generally expected that segments expressed on the surface of a virion will have higher rates of evolutionary change than those that only have internal functions, because the former have roles in immune evasion. We showed that VP1 has experienced strong positive selection which increased its divergence and decreased polymorphisms. As a result, the rate of change derived from the polymorphic data should be slower for VP1, because strong selection makes the population less polymorphic (Table 2).

### Lack of recombination in the current viral isolates

Only one recombination event identified in 401 viral isolates seems inconsistent with the general notion that enteroviruses frequently recombine. Nevertheless, except for the year 2000, there was only one dominant lineage in each epidemic season. The chance of recombination between different lineages was, thus, reduced. In addition, levels of nucleotide variations within lineages were < 1%. Most methods for detecting recombinations perform poorly at this level of divergence [47]. It is, then, not surprising that only one recombined viral strain was identified. It is worth pointing out that even though there might be a small chance of unidentified recombinants, our conclusions might not or only minimally be affected for a couple of reasons. First, recombination within a lineage does not change the numbers of synonymous and nonsynonymous mutations or the N/S ratio in a circulating viral population, and, thus, will not influence the observed nucleotide diversities and simulation results. Second, in the analysis of the TMRCA and mutation rate, BEAST assumes no recombination and accounts for deviations from this in the data with multiple mutations. As a result, it may overestimate genetic diversities and mutation rates (Table 5) with the existence of recombinations [48]. While the effect of recombinations on TMRCA estimations is still being debated [49-51], TMRCAs from different regions were in generally agreement with each other. Thus, unless different genomic regions have similar patterns of recombination, the existence of unidentified recombinations, if any, should not largely affect our conclusions.

## Conclusions

Contrasting patterns of polymorphisms and divergences between different protein coding regions suggest that different evolutionary forces have been dominant in different regions of the EV71 genome. The results of a computer simulation demonstrated a significant excess of amino acid replacements in the VP1 region of local viral isolates which implies a possible role of adaptive evolution. Among different species of HEV-A, exchange of the P1 domain was frequently observed. We propose that positive selection followed by domain shuffling contributed to the emergence of new HEV-A genotypes. Consistent patterns of an increase in viral diversity in the epidemic peak followed by severe diversity reduction were observed in different epidemic seasons. In addition, the viruses sampled in local successive epidemic seasons were not sister to each other, indicating that annual outbreaks of EV71 were due to genetically distinct lineages. It is likely that the importation of EV71 from surrounding areas contributed to local outbreaks.

## Methods

### Viral isolation, reverse-transcription polymerase chain reaction (RT-PCR), and sequencing

Throat swabs, rectal swabs, or cerebrospinal fluid (CSF) were obtained from patients who had clinical manifestations of HFMD or herpangina, viral exanthema, viral meningitis, encephalitis, polio-like syndrome, or cardiopulmonary failure with CNS involvement at Chang Gung Children's Hospital, Taoyuan, Taiwan. Viral isolation was performed by inoculating human embryonic fibroblast (MRC-5), LLC-MK2, HEp-2, and RD cell cultures with the above specimens. Once the enteroviral cytopathic involvement exceeded 50% of the cell monolayer, cells were scraped off and subjected to indirect fluorescent antibody staining with a panenteroviral antibody (catalog no. 3360, Chemicon International, Temecula, CA) to confirm the enterovirus. These isolates were subsequently identified as EV71 by an immunofluorescence assay if they had simultaneously positive immunofluorescence reactions to both EV71-specific antibodies (catalog nos. 3323 and 3324, Chemicon International). EV71 isolates were found in 395 EV71 patients from 1998 to 2003. Six cases were identified as EV71 by the immunofluorescence assay which turned out to be the coxsackievirus A16-like virus based on their VP1 sequences (see "Results"). Therefore, 401 isolates were collected and processed for this study. The demographic data, clinical severity, the year of isolation, and the site of the specimens are listed in Additional file 1, Table S1.

EV71 isolates were stored at -80°C and re-cultured in rhadomyosarcome (RD) cells. Viral RNA was extracted from RD cells using the QIAamp Viral RNA Mini Kit (Qiagen, Valencia, CA). A one-step RT-PCR was performed using the Titan One Tube RT-PCR System (Roche Diagnostics, Indianapolis, IN). Additional file 4, Table S2, lists the primers and PCR conditions for different genomic regions of EV71, including the 5'UTR, VP1, 2A, and 3C. After amplification of the viral RNA, the PCR products were purified using a High Pure PCR Product Purification Kit (Roche Diagnostics, Indianapolis, IN). Cycle sequencing was performed using an ABI Prism BigDye^® ^Terminator cycle sequencing kit and ABI Prism 3730 DNA sequencer (Applied Biosystems, Foster City, CA).

### Sequence analysis

Sequences were aligned using ClustalW [52] and edited manually. Watterson's estimator of nucleotide diversity (θ) was calculated using the custom C++ software based on the libsequence package [53]. For protein coding regions, mutations were classified as either synonymous or nonsynonymous, and from these we calculated the nucleotide diversity for synonymous (θ_s_) and nonsynonymous (θ_a_) sites. Numbers of synonymous substitutions per synonymous site (Ks) and numbers of nonsynonymous substitutions per nonsynonymous site (Ka) were calculated according to the modified Nei and Gojobori method [54,55]. This method has two advantages. First, when sequence divergence is high, the number of inapplicable cases is much lower than that with other methods [56-58]. Second, its calculations take the transitional/transversional (TS/TV) mutation bias into account. Because of a high viral mutation rate, nucleotide substitutions in synonymous sites were saturated (Ks > 1, see "Results"). In addition, mutations in EV71 were highly biased toward transitions. In the Ka and Ks calculations, TS/TV ratios of different genomic segments were determined based on the results of model selection (see below).

### Recombination and phylogenetic reconstruction

Three methods, including the method of Sawyer [21] implemented in GENECONV 1.81a http://www.math.wustl.edu/~sawyer, the homoplasy test [20] implemented in START [59], and GARD (Genetic Algorithm Recombination Detection) [22] available online http://Datamonkey.org[60] were applied to detect possible recombination events between and within EV71 genotypes. The first is suitable for 5%~20% nucleotide differences which was the range of divergence between different genotypes of EV71, and the second is particularly useful for < 5% differences, which was the level of divergence within genotypes. The numbers of permutations were 10,000 for GENECONV and 1000 for the homoplasy test. The significance level used was 5%. To visualize the recombination break points, the bootscan program implemented in SIMPLOT [61] was applied. We used a sliding window size of 200 nucleotides with a step size of 20 nucleotides. TS/TV ratios were estimated from the sequences. NJ trees were estimated using F84 distances, and bootstrap support values were obtained from 500 replicates. Bootstrap values were then plotted along the position of the genome. A recombination event was suggested when high levels of phylogenetic relatedness (> 70% bootstrap values) between the query group and more than one reference group in different genomic regions were observed. Bootscan was further applied to detect possible recombinations between different species of HEV-A. All settings were the same, except that a sliding window of 400 nucleotides was used.

The nucleotide substitution model for phylogenetic reconstruction was determined by the Akaike information criterion (AIC) [62] using Modeltest 3.06 [63] and is listed in the online Additional file 5. The neighbor-joining (NJ) tree was constructed using PAUP* vers. 4.0b10. The maximum-likelihood (ML) analysis was conducted using PhyML 3.0 online [64] with the starting tree derived from the NJ method, and the NNI topology search option was used. Bayesian inference (BI) was performed using MrBayes vers. 3.1.2 [65]. Analyses were initiated with random starting trees, and Metropolis-coupled Markov chain Monte Carlo (MCMC) analyses were run for 4 × 10^6 ^generations and sampled every 100 generations. The first 4000 trees were excluded while the remaining 36000 trees were retained to compute a 50% majority-rule consensus tree. Human enterovirus (HEV)-A, including coxsackievirus A2 (CA2), CA3, CA4, CA5, CA6, CA7, CA8, CA10, CA12, CA14, and CA16, and the prototype EV71 BrCr-CA-70 (EV71- A) were also included in the tree construction with enterovirus 90, AY773285 and AB192877, as the outgroups. Bootstrap replications for nodal support were 500 for the ML and 1000 for the NJ analyses. To further trace the origin of the viruses, we included and aligned EV71 sequences from surrounding areas in the 1990 s to 2003 [11,13,15,17,18]. Phylogenetic relationships of these sequences were constructed using the NJ method with 1000 bootstrap replications.

### Computer simulation

To test whether nucleotide substitutions in different genomic regions followed the neutral mutation hypothesis, i.e., variations within populations and divergences between species were both mainly due to neutral or nearly neutral mutations, we carried out the following computer simulations. (1) Using the consensus sequence of EV71-B4 or -C2 as a starting point, the position of change in the sequence was randomly chosen. (2) For different protein coding regions, the change was then determined to be either synonymous (S) or nonsynonymous (N) according to the N/S ratio of the polymorphism. Stop codon changes were not counted. (3) The change was then assigned as a transition or transversion based on the results of the model selection (see "Additional file 5"). (4) The process was repeated until the Ks value equaled the average Ks value between EV71 and HEV-A. (5) The simulation was repeated 10,000 times. The results were tallied for each replicate, and distributions were used to determine the *p *value for the observed Ka and Ka/Ks values.

### Codon-based maximum likelihood ratio test for positive selection

To detect the signal of positive selection, two approaches were applied. Natural selection can be detected by comparing Ks with Ka. Ka > Ks indicates positive selection, whereas Ka < Ks indicates negative selection. Several methods were developed to test for positive selection that acts on given branches (branch method), on a subset of sites (site method), or at individual sites along specific branches (branch-site method) [66-68]. To identify the putative amino acid residues under positive selection and to estimate the strength of the selection, two pairs of likelihood-based models implemented in the PAML [69] package was used. These models allow the Ka/Ks ratio to vary among sites. The first pair include M1a (nearly neutral) and M2a (positive selection) [70], while the second pair includes M7 (beta) and M8 (beta&ω) [71]. We also used branch-site test 2 to detect positive selection of the lineages of interest [68]. To test whether different protein coding regions are under similar selection regimes, we used dNdSDistributionComparison.bf implemented in HyPhy 2.0 [22]. This program divides codons into four categories, two negatively selected (Ka < Ks), one neutral (Ka = Ks), and one positively selected (Ka > Ks), and tests whether different genes have similar (1) strengths of positive selection, (2) proportions of sites under positive selection, and (3) distributions for all sites.

### Estimates of population dynamics and evolutionary rates

The evolutionary dynamics of EV71 were estimated using an established Bayesian Markov chain Monte Carlo (MCMC) approach [72,73] that incorporates the exact month of viral sampling. This approach estimates the rate of nucleotide changes and the dynamics of population genetic diversity through time [74]. In addition, the time to the most recent common ancestor (TMRCA) of viruses isolated from each epidemic season was also estimated. Because EV71-C2 was mainly recovered from the 1998 epidemic season, the mutation rate and population dynamic estimated were primarily based on EV71-B4 collected from 1998 to 2003.

The analyses were performed using the SRD06 [75] model of nucleotide substitution by BEAST v1.4.8. Both strict and relaxed (uncorrelated lognormal) molecular clock model [76] were used and the results are essentially the same (Bayes factor = 0.18). For population demographic, three models, including constant size, exponential growth, and Bayesian skyline plot (BSP) [74], were tested and the BSP performed significantly better than the other two (Bayes factor > 20). In cases where sequences were sampled in the same month with higher than 99% nucleotide identity, four of the sequences were randomly selected. This resulted in a dataset of 145 EV71-B4 and 22 EV71-C2 sequences (listed in the "Additional file 5"). For each genetic segment, we used two independent runs with 2 × 10^7 ^MCMC steps, of which the first 10% was discarded as burn-in. The results were compared to confirm that both were sampling the same distribution and then were combined. The log files were checked using TRACER, and the effective sample size for each parameter was found to exceed 200. Since the time span in this study was less than 6 years, in order to test whether the sequences contained a sufficient signal to estimate evolutionary rates and divergence time, two approaches were applied. First, we included 108 and 48 dated B4 and C2 sequences, respectively, from other areas between the 1990 s and 2003 [11,13,15,17,18] for the TMRCA estimation (listed in the "Additional file 5"). Second, we randomized the sampling dates of our sequences and repeated the BEAST analysis [77]. In the second analysis only Taiwanese sequences were used. The 95% HPDs of real and randomized data were compared to test whether the distributions overlapped.

The evolutionary rate was also estimated for EV71-B4 by a linear regression of the genetic distance from the oldest isolate versus the time of isolation. For each protein coding region, two separate analyses were conducted: one for all nucleotide positions (representative of both synonymous and nonsynonymous changes) and a second one for only synonymous changes. The former was calculated based on the best model suggested by Modeltest and the latter was calculated according to the modified Nei and Gojobori method.

## Authors' contributions

LYC, SRS, and LMH designed the study. TYL, SRS, and LYC collected the samples. KCT and LYC performed the experiments. HYW, KCT, CHH and GWC analyzed the data. LMH, SRS, and LYC provided reagents and chemicals. HYW and LYC wrote the paper. All authors read and approved the final manuscript.

## Supplementary Material

Additional file 1 **List of viral isolates used in the molecular genetics of enterovirus (EV) 71**. Table S1. Click here for file

Additional file 2 **Identification of recombined regions in current viral isolates**. Figure S1. Click here for file

Additional file 3 **Neighbor-joining trees showing the phylogenetic relationships among human enterovirus (HEV)-A and enterovirus (EV) 71 isolates**. Figure S2. Click here for file

Additional file 4 **Primers, RT-PCR, and PCR conditions for VP1, 2A, 3C, and the 5' untranslated region (UTR)**. Table S2. Click here for file

Additional file 5 **Supplemental information**. Appendix. Click here for file
