# Supplementary material for: Inferring nonneutral evolution from contrasting patterns of polymorphisms and divergences in different protein coding regions of enterovirus 71 circulating in Taiwan during 1998-2003
Source: BMC Evol Biol. 2010 Sep 25;10:294. doi: 10.1186/1471-2148-10-294 (PMC2958165; doi:10.1186/1471-2148-10-294)
Supplement: Additional file 2 — Identification of recombined regions in current viral isolates. Figure S1. [file 1471-2148-10-294-S3.DOC]

Figure S1. Identification of recombined regions in current viral isolates. The upper panel shows sequenced regions of EV71. The lower panel shows the results from bootscan analysis in which lineage 10, TW-2051-98, was queried against other lineages using a window size of 400 bp with step size of 20 bp.
