# Supplementary material for: Inferring nonneutral evolution from contrasting patterns of polymorphisms and divergences in different protein coding regions of enterovirus 71 circulating in Taiwan during 1998-2003
Source: BMC Evol Biol. 2010 Sep 25;10:294. doi: 10.1186/1471-2148-10-294 (PMC2958165; doi:10.1186/1471-2148-10-294)
Supplement: Additional file 4 — Primers, RT-PCR, and PCR conditions for VP1, 2A, 3C, and the 5' untranslated region (UTR). Table S2. [file 1471-2148-10-294-S2.DOC]

Table S2. Primers, RT-PCR, and PCR conditions for VP1, 2A, 3C, and the 5’untranslated region (UTR)

| Gene | Primer sequence (5’ to 3’) | Nucleotide position* | Polarity | PCR condition |
| --- | --- | --- | --- | --- |
| VP1 | 5’GCAGCCCARAAGAAYTTYAC | 2370 ~ 2389 | Sense | 50^O^C for 30min, 94^O^C for 2min; 94^O^C 30sec, 52^O^C 30sec, 68^O^C 1min, for 35 repeats; 68^O^C for 7min |
|  | 5’GAGCTRTCYTCCCAVACRAG | 3442~ 3423 | Antisense |
| 2A | 5’GTCAGRGCGTGGATACCTCG | 3213~ 3232 | Sense | 50^O^C for 30min, 94^O^C for 2min; 94^O^C 30sec, 53^O^C 30sec, 68^O^C 1min, for 35 repeats; 68^O^C for 7min |
|  | 5’GCTTCAACTTCYCTKTCYGAYAC | 3862~3843 | Antisense |
| 3C | 5’ TTYCARGGWGCDTAYTCY | 5319~ 5338 | Sense | 50^O^C for 30min, 94^O^C for 2min; 94^O^C 30sec, 55^O^C 30sec, 68^O^C 1min, for 35 repeats; 68^O^C for 7min |
|  | 5’ TGATGTTCAACCTGCCAGTTTCTTT | 5991~ 5967 | Antisense |
|  | 5’ AYCCAYTGGATCTCWCCTTG | 5956~ 5937 | Antisense |
| 5’UTR | 5’AGYAGGYRTRRCRCRCCAG | 148 ~ 167 | Sense | 50^O^C for 30min, 94^O^C for 2min; 94^O^C 30sec, 55^O^C 30sec, 72^O^C 1min, for 35 repeats; 72^O^C for 7min |
|  | 5’ RGAYACYTGTGARCCCAT | 761 ~ 744 | Antisense |
| 5’UTR | 5’ GRWAYCYTTGTRCGCCTGTT | 66 ~ 85 | Sense | 50^O^C for 30min, 94^O^C for 2min; 94^O^C 30sec, 53^O^C 30sec, 72^O^C 1min, for 35 repeats; 72^O^C for 7min |
|  | 5’ ACAGGRTTHGCAAAYTTRTC | 920 ~ 901 | Antisense |

R=A /G; Y=C/T; M=A/C; K=G/T; S=C/G; W=A/T; H=A/C/T; B=C/G/T; V=A/C/G; D=A/G/T; N=A/C/G/T; * nucleotide position in reference to the EV71 2272/98/TW strain
