# Supplementary material for: Inferring nonneutral evolution from contrasting patterns of polymorphisms and divergences in different protein coding regions of enterovirus 71 circulating in Taiwan during 1998-2003
Source: BMC Evol Biol. 2010 Sep 25;10:294. doi: 10.1186/1471-2148-10-294 (PMC2958165; doi:10.1186/1471-2148-10-294)
Supplement: Additional file 5 — Supplemental information. Appendix. [file 1471-2148-10-294-S5.DOC]

**Supplemental Information**

Nucleotide substitution models for the phylogenetic reconstructions of different genomic regions are listed below. These models were determined by the Akaike Information Criterion (AIC) [1] using Modeltest 3.06 [2].

**VP1**

Model selected: GTR+I+G

Base frequencies:

freqA = 0.2928

freqC = 0.2676

freqG = 0.2117

freqT = 0.2279

Substitution model:

Rate matrix

R(a) [A-C] = 3.9455

R(b) [A-G] = 9.6576

R(c) [A-T] = 2.6217

R(d) [C-G] = 2.2116

R(e) [C-T] = 12.9485

R(f) [G-T] = 1.0000

Among-site rate variation

Proportion of invariable sites (I) = 0.1967

Variable sites (G)

Gamma distribution shape parameter = 0.5940

**2A**

Model selected: SYM+I+G

Base frequencies:

Equal frequencies

Substitution model:

Rate matrix

R(a) [A-C] = 2.8256

R(b) [A-G] = 18.6307

R(c) [A-T] = 4.2585

R(d) [C-G] = 1.3887

R(e) [C-T] = 42.6493

R(f) [G-T] = 1.0000

Among-site rate variation

Proportion of invariable sites (I) = 0.3765

Variable sites (G)

Gamma distribution shape parameter = 0.5110

**3C**

Model selected: TVM+G

Base frequencies:

freqA = 0.2637

freqC = 0.2639

freqG = 0.2215

freqT = 0.2509

Substitution model:

Rate matrix

R(a) [A-C] = 0.7692

R(b) [A-G] = 21.5787

R(c) [A-T] = 2.9060

R(d) [C-G] = 1.1049

R(e) [C-T] = 21.5787

R(f) [G-T] = 1.0000

Among-site rate variation

Proportion of invariable sites = 0

Variable sites (G)

Gamma distribution shape parameter = 0.1702

**5'UTR**

Model selected: TVMef+I+G

Base frequencies:

Equal frequencies

Substitution model:

Rate matrix

R(a) [A-C] = 1.7220

R(b) [A-G] = 15.3550

R(c) [A-T] = 4.2437

R(d) [C-G] = 0.9375

R(e) [C-T] = 15.3550

R(f) [G-T] = 1.0000

Among-site rate variation

Proportion of invariable sites (I) = 0.3410

Variable sites (G)

Gamma distribution shape parameter = 0.6271

**Recombination analysis**

Potential recombinations among EV71 isolates were determined by the bootscan strategy implemented in the Simplot computer software [3]. In this analysis, a sliding window of 400 nucleotides was moved with a step size of 20 nucleotides at a time. In each window, Neighbor-joining trees based on F84 distances were constructed with 500 bootstrap replications. The bootstrap values were then plotted along the position of the genome. A recombination event was suggested when high levels of phylogenetic relatedness between a query sequence and more than one reference sequence in different genomic regions were observed. In the analysis, genome sequences of EV71 and HEV-A sequences used for recombination analysis were as follows.

EV71-B (U22522, AF352027, AF316321)

EV71-A (U22521)

EV71-C1 (DQ452074)

EV71-C2 (AF119795, AF176044)

EV71-C4 (AF302996, AY465356)

CA16-like (AF177911, AY790926)

CA2 (AY421760)

CA3 (AY421761)

CA4 (AY421762)

CA5 (AY421763)

CA6 (AY421764)

CA7 (AY421765)

CA8 (AY421766)

CA10 (AY421767)

CA12 (AY421768)

CA14 (AY421769)

CA16 (U05876)

**List of viral strains used in the phylogenetic tree construction shown in Fig. S1.**

TW-1370-98

TW-1465-98

TW-1496-98

TW-1507-98

TW-1539-98

TW-1547-98

TW-1602-98

TW-1624-98

TW-1663-98

TW-1692-98

TW-1700-98

TW-1707-98

TW-1708-98

TW-1733-98

TW-1738-98

TW-1747-98

TW-1761-98

TW-1788-98

TW-1792-98

TW-1851-98

TW-1859-98

TW-1875-98

TW-1909-98

TW-1910-98

TW-1924-98

TW-1931-98

TW-1933-98

TW-1935-98

TW-1944-98

TW-1970-98

TW-2005-98

TW-2025-98

TW-2051-98

TW-2055-98

TW-2057-98

TW-2060-98

TW-2077-98

TW-2086-98

TW-2100-98

TW-2101-98

TW-2155-98

TW-2172-98

TW-2200-98

TW-2239-98

TW-2254-98

TW-2286-98

TW-2350-98

TW-2414-98

TW-2418-98

TW-2433-98

TW-2459-98

TW-2495-98

TW-2543-98

TW-2553-98

TW-2651-98

TW-2678-98

TW-2733-98

TW-2740-98

TW-2743-98

TW-2799-98

TW-2849-98

TW-2861-98

TW-2869-98

TW-2880-98

TW-2898-98

TW-2911-98

TW-2917-98

TW-2922-98

TW-2938-98

TW-2972-98

TW-2973-98

TW-2981-98

TW-2982-98

TW-2988-98

TW-3014-98

TW-3026-98

TW-3034-98

TW-3074-98

TW-3124-98

TW-3126-98

TW-3130-98

TW-3170-98

TW-3180-98

TW-3241-98

TW-3283-98

TW-3296-98

TW-3298-98

TW-3339-98

TW-3411-98

TW-3436-98

TW-3445-98

TW-3480-98

TW-3481-98

TW-3483-98

TW-3485-98

TW-3510-98

TW-3535-98

TW-3550-98

TW-3584-98

TW-3592-98

TW-3620-98

TW-3702-98

TW-3712-98

TW-3724-98

TW-4106-98

TW-4215-98

TW-4368-98

TW-4375-98

TW-4510-98

TW-4866-98

TW-4925-98

TW-4931-98

TW-4940-98

TW-5056-98

TW-5390-98

TW-1394-99

TW-1691-99

TW-3351-99

TW-4711-99

TW-4912-99

TW-645-00

TW-1155-00

TW-1184-00

TW-1743-00

TW-1769-00

TW-1801-00

TW-1804-00

TW-1995-00

TW-1996-00

TW-2006-00

TW-2027-00

TW-2179-00

TW-2188-00

TW-2200-00

TW-2238-00

TW-2242-00

TW-2278-00

TW-2293-00

TW-2387-00

TW-2404-00

TW-2448-00

TW-2473-00

TW-2586-00

TW-2588-00

TW-2632-00

TW-2700-00

TW-2708-00

TW-2720-00

TW-2760-00

TW-2779-00

TW-2784-00

TW-2797-00

TW-2847-00

TW-3123-00

TW-3131-00

TW-3236-00

TW-3307-00

TW-3404-00

TW-3594-00

TW-3704-00

TW-3857-00

TW-3882-00

TW-3970-00

TW-4138-00

TW-4156-00

TW-4279-00

TW-4282-00

TW-4507-00

TW-4527-00

TW-4535-00

TW-4566-00

TW-4568-00

TW-4664-00

TW-4729-00

TW-4741-00

TW-5110-00

TW-5185-00

TW-5365-00

TW-5405-00

TW-5426-00

TW-5447-00

TW-645-01

TW-725-01

TW-1069-01

TW-1280-01

TW-1377-01

TW-1451-01

TW-1470-01

TW-1482-01

TW-1484-01

TW-1508-01

TW-1583-01

TW-1652-01

TW-1660-01

TW-1662-01

TW-1690-01

TW-1854-01

TW-1894-01

TW-1940-01

TW-2002-01

TW-2065-01

TW-2089-01

TW-2210-01

TW-2211-01

TW-2220-01

TW-2276-01

TW-2307-01

TW-2317-01

TW-2325-01

TW-2330-01

TW-2331-01

TW-2348-01

TW-2359-01

TW-2459-01

TW-2466-01

TW-2467-01

TW-2489-01

TW-2490-01

TW-2525-01

TW-2582-01

TW-2587-01

TW-2588-01

TW-2608-01

TW-2666-01

TW-2676-01

TW-2679-01

TW-2707-01

TW-2736-01

TW-2743-01

TW-2790-01

TW-2803-01

TW-2812-01

TW-2815-01

TW-2823-01

TW-2844-01

TW-2857-01

TW-2915-01

TW-2935-01

TW-3118-01

TW-3137-01

TW-3147-01

TW-3151-01

TW-3161-01

TW-3166-01

TW-3171-01

TW-3175-01

TW-3194-01

TW-3318-01

TW-3381-01

TW-3389-01

TW-3398-01

TW-3439-01

TW-3459-01

TW-3482-01

TW-3499-01

TW-3523-01

TW-3570-01

TW-3647-01

TW-3684-01

TW-3694-01

TW-3797-01

TW-3800-01

TW-3802-01

TW-3806-01

TW-3807-01

TW-3815-01

TW-3830-01

TW-3894-01

TW-3909-01

TW-3911-01

TW-3921-01

TW-4021-01

TW-4048-01

TW-4064-01

TW-4094-01

TW-4103-01

TW-4137-01

TW-4141-01

TW-4173-01

TW-4294-01

TW-4331-01

TW-4356-01

TW-4399-01

TW-4411-01

TW-4438-01

TW-4451-01

TW-4486-01

TW-4530-01

TW-4625-01

TW-4662-01

TW-4681-01

TW-4687-01

TW-4752-01

TW-4891-01

TW-4924-01

TW-4954-01

TW-4968-01

TW-4983-01

TW-5026-01

TW-5074-01

TW-5078-01

TW-5089-01

TW-5337-01

TW-5373-01

TW-5422-01

TW-5461-01

TW-5472-01

TW-5491-01

TW-5514-01

TW-5531-01

TW-5536-01

TW-5567-01

TW-5580-01

TW-5612-01

TW-5633-01

TW-5686-01

TW-5824-01

TW-5825-01

TW-5904-01

TW-6126-01

TW-6165-01

TW-6173-01

TW-6205-01

TW-6211-01

TW-6213-01

TW-6326-01

TW-6349-01

TW-6528-01

TW-6540-01

TW-146-02

TW-270-02

TW-593-02

TW-616-02

TW-655-02

TW-690-02

TW-748-02

TW-855-02

TW-916-02

TW-1127-02

TW-1727-02

TW-1889-02

TW-1891-02

TW-1893-02

TW-1894-02

TW-1922-02

TW-2034-02

TW-2045-02

TW-2071-02

TW-2190-02

TW-2196-02

TW-2699-02

TW-2792-02

TW-2822-02

TW-2885-02

TW-3074-02

TW-3389-02

TW-3417-02

TW-3506-02

TW-3509-02

TW-3538-02

TW-3552-02

TW-3554-02

TW-3686-02

TW-3716-02

TW-3804-02

TW-3821-02

TW-3881-02

TW-3895-02

TW-3909-02

TW-3929-02

TW-4005-02

TW-4151-02

TW-5086-02

TW-5533-02

TW-5611-02

TW-5634-02

TW-5762-02

TW-5817-02

TW-5837-02

TW-5843-02

TW-5861-02

TW-5863-02

TW-6006-02

TW-6103-02

TW-6269-02

TW-6280-02

TW-6287-02

TW-6301-02

TW-6367-02

TW-6368-02

TW-6382-02

TW-6407-02

TW-6468-02

TW-6736-02

TW-70146-03

TW-70150-03

TW-70167-03

TW-70233-03

TW-991-03

TW-1487-03

TW-70576-03

BrCr (genotype A)

CA2

CA3

CA4

CA5

CA6

CA7

CA8

CA10

CA12

CA14

CA16

enterovirus/90/F950027

enterovirus/90/CAM1956

**List of viral strains used in the evolutionary dynamic estimation shown in Fig. 1 and Table 5. Two digits at the end are year of sampling.**

**Sequences from Taiwan**

**Genotype B4**

TW-645-00

TW-725-01

TW-1069-01

TW-1470-01

TW-1583-01

TW-1940-01

TW-2089-01

TW-2211-01

TW-2467-01

TW-2679-01

TW-3166-01

TW-3389-01

TW-3523-01

TW-3909-01

TW-4064-01

TW-4356-01

TW-4411-01

TW-4451-01

TW-4752-01

TW-4891-01

TW-4924-01

TW-4954-01

TW-4968-01

TW-5026-01

TW-5337-01

TW-5373-01

TW-5422-01

TW-5461-01

TW-5472-01

TW-5491-01

TW-5514-01

TW-5531-01

TW-5536-01

TW-5567-01

TW-5580-01

TW-5612-01

TW-5633-01

TW-5686-01

TW-5824-01

TW-5825-01

TW-5904-01

TW-6126-01

TW-6165-01

TW-6211-01

TW-6326-01

TW-6349-01

TW-6528-01

TW-146-02

TW-270-02

TW-593-02

TW-616-02

TW-655-02

TW-690-02

TW-748-02

TW-855-02

TW-916-02

TW-1127-02

TW-1727-02

TW-1891-02

TW-2034-02

TW-2071-02

TW-2196-02

TW-2699-02

TW-2792-02

TW-2822-02

TW-2885-02

TW-3389-02

TW-3417-02

TW-3509-02

TW-3538-02

TW-3552-02

TW-3554-02

TW-3716-02

TW-3804-02

TW-3881-02

TW-4005-02

TW-4151-02

TW-5086-02

TW-5533-02

TW-5611-02

TW-5762-02

TW-5837-02

TW-5843-02

TW-5861-02

TW-6280-02

TW-6301-02

TW-6367-02

TW-6468-02

TW-6736-02

TW-70146-03

TW-70150-03

TW-70167-03

TW-70233-03

TW-991-03

TW-1487-03

TW-70576-03

TW-1184-00

TW-1743-00

TW-1804-00

TW-1996-00

TW-2188-00

TW-3131-00

TW-3236-00

TW-3307-00

TW-3404-00

TW-3704-00

TW-3857-00

TW-4138-00

TW-4156-00

TW-4279-00

TW-4282-00

TW-4507-00

TW-4535-00

TW-4566-00

TW-4664-00

TW-5110-00

TW-5185-00

TW-5365-00

TW-5405-00

TW-5426-00

TW-645-01

TW-1280-01

TW-2276-01

TW-5089-01

TW-4912-99

TW-1155-00

TW-1995-00

TW-2238-00

TW-2242-00

TW-3594-00

TW-3882-00

TW-3970-00

TW-4527-00

TW-4568-00

TW-4729-00

TW-4741-00

TW-5447-00

TW-3351-99

TW-4711-99

TW-1370-98

TW-2495-98

TW-2678-98

TW-2898-98

TW-3339-98

TW-1394-99

**Genotype C2**

TW-1465-98

TW-1747-98

TW-1851-98

TW-2005-98

TW-2086-98

TW-2101-98

TW-3130-98

TW-3180-98

TW-3436-98

TW-3485-98

TW-3550-98

TW-4106-98

TW-4368-98

TW-4375-98

TW-4510-98

TW-4866-98

TW-4925-98

TW-4931-98

TW-4940-98

TW-5056-98

TW-5390-98

TW-1691-99

**Sequences from surrounding areas**

**Genotype B4**

9924-SYD-00

9906-SYD-01

2999-SYD-01

3242-SYD-01

2573-SYD-01

9911-SYD-01

9912-SYD-01

9902-SYD-01

9901-SYD-01

9917-SYD-01

9929-SYD-00

9923-SYD-01

2287-SYD-01

2790-SYD-01

5546-SIN-00

2267-SIN-01

5926-SIN-00

2120-SIN-01

5556-SIN-00

5859-SIN-00

5769-SIN-00

5869-SIN-00

5881-SIN-00

0042-MAA-00

SB0635-SAR-00

2027-SIN-01

0066-MAA-00

0467-MAA-00

2123-SIN-01

2386-SIN-01

0431-MAA-00

0778-MAA-00

0627-MAA-99

0919-MAA-99

5536-SIN-00

CN04104-SAR-00

S21082-SAR-00

CN062334-SAR-00

SB1191-SAR-00

CN0942-SAR-00

S40201-SAR-00

SB1647-SAR-00

CN9502-SAR-00

SB2864-SAR-00

S12502-SAR-00

S12172-SAR-00

S2861-SAR-00

2266-SYD-00

9918-SYD-00

1067-Yamagata-00

934-Yamagata-00

980-Yamagata-00

1084-Yamagata-00

1141-Yamagata-00

962-Yamagata-00

738-Yamagata-00

739-Yamagata-00

786-Yamagata-00

0615-MAA-99

0898-MAA-97

03300-MAA-97

0343-MAA-97

0128-MAA-97

0091-MAA-97

0175-MAA-97

0414-MAA-97

0815-MAA-00

5511-SIN-00

2419-Yamagata-03

2542-Yamagata-03

2716-Yamagata-03

2972-Yamagata-03

2933-Yamagata-03

2934-Yamagata-03

0036-MAA-97

0903-MAA-97

0884-MAA-97

0473-MAA-97

0897-MAA-97

0899-MAA-97

0245-MAA-97

04716-MAA-97

3799-SIN-98

0870-MAA-97

MY860-3-SAR-97

MY104-9-SAR-97

MY755-3-SAR-97

MY821-3-SAR-97

MY6-2-SAR-97

4284-SIN-98

4350-SIN-98

3526-SIN-98

3F-AUS-3-99

MY16-1-SAR-97

4F-AUS-4-99

25M-AUS-2-99

18F-AUS-6-99

1M-AUS-3-99

11F-AUS-6-99

26M-AUS-2-99

17M-AUS-5-99

19M-AUS-6-99

20F-AUS-6-99

14F-AUS-9-99

MY21-2-SAR-97

10M-AUS-6-99

12F-AUS-6-99

16F-AUS-6-99

**Genotype C2**

9166-TX-89

9541-TX-89

9323-TX-89

9873-NM-89

8495-VA-88

1411-CA-90

2623-AUS-86

9837-WA-89

0926-OR-91

0925-OR-91

2246-NY-87

2245-NY-87

7238-AK-87

7237-AK-87

0916-MA-87

0915-MA-87

2583-CAN-91

2261-CA-91

2640-AUS-95

0443-TX-91

2006-CT-94

2263-CA-94

2254-NY-94

2251-NY-93

2253-NY-94

2037-MD-95

2132-VA-95

1873-CT-93

9243-OK-89

2262-CA-92

1924-AZ-94

2264-CA-94

9718-TX-89

9978-TX-89

0390-TX-91

0359-TX-90

1997-NC-94

2007-CT-94

1919-NM-94

0756-MAA-97

0667-CHN-87

2286-TX-97

2381-MA-97

2814-MO-98

2355-OK-97

2642-AUS-95

2644-AUS-95

2641-AUS-95

1. Posada D, Buckley TR: **Model selection and model averaging in phylogenetics: advantages of Akaike Information Criterion and Bayesian approaches over likelihood ratio tests.** *Syst Biol* 2004, **53:**793-808.

2. Posada D, Crandall KA: **MODELTEST: testing the model of DNA substitution.** *Bioinformatics* 1998, **14:**817-818.

3. Lole KS, Bollinger RC, Paranjape RS, Gadkari D, Kulkarni SS, Novak NG, Ingersoll R, Sheppard HW, Ray SC: **Full-length human immunodeficiency virus type 1 genomes from subtype C-infected seroconverters in India, with evidence of intersubtype recombination.** *J Virol* 1999, **73:**152-160.
